# Differential Virulence of *Candida glabrata* Glycosylation Mutants*[Fn FN2]

**DOI:** 10.1074/jbc.M113.478743

**Published:** 2013-05-28

**Authors:** Lara West, Douglas W. Lowman, Héctor M. Mora-Montes, Sarah Grubb, Craig Murdoch, Martin H. Thornhill, Neil A.R. Gow, David Williams, Ken Haynes

**Affiliations:** From the ‡ Department of Microbiology, Imperial College London, London, SW7 2AZ, United Kingdom,; the § Department of Surgery, Quillen College of Medicine, East Tennessee State University, Johnson City, Tennessee 37614,; ¶ AppRidge International, LLC, Jonesborough, Tennessee 37659-0266,; the ‖ School of Medical Sciences, Institute of Medical Sciences, University of Aberdeen, Foresterhill, Aberdeen AB25 2ZD, United Kingdom,; the ** Departamento de Biología, División de Ciencias Naturales y Exactas, Universidad de Guanajuato, Noria Alta s/n, Col. Noria Alta, Guanajuato, Gto. 36050, México,; the ‡‡ University of Sheffield School of Clinical Dentistry, Sheffield, S10 2TA, United Kingdom, and; §§ Biosciences, College of Life and Environmental Sciences, University of Exeter, Exeter, EX4 4QD, United Kingdom

**Keywords:** Candida albicans, Cell Wall, Fungi, Host-Pathogen Interactions, Virulence Factors

## Abstract

The fungus *Candida glabrata* is an important and increasingly common pathogen of humans, particularly in immunocompromised hosts. Despite this, little is known about the attributes that allow this organism to cause disease or its interaction with the host immune system. However, in common with other fungi, the cell wall of *C. glabrata* is the initial point of contact between the host and pathogen, and as such, it is likely to play an important role in mediating interactions and hence virulence. Here, we show both through genetic complementation and polysaccharide structural analyses that *C. glabrata ANP1, MNN2,* and *MNN11* encode functional orthologues of the respective *Saccharomyces cerevisiae* mannosyltransferases. Furthermore, we show that deletion of the *C. glabrata* Anp1, Mnn2, and Mnn11 mannosyltransferases directly affects the structure of the fungal *N*-linked mannan, in line with their predicted functions, and this has implications for cell wall integrity and consequently virulence. *C. glabrata anp1* and *mnn2* mutants showed increased virulence, compared with wild-type (and *mnn11*) cells. This is in contrast to *Candida albicans* where inactivation of genes involved in mannan biosynthesis has usually been linked to an attenuation of virulence. In the long term, a better understanding of the attributes that allow *C. glabrata* to cause disease will provide insights that can be adopted for the development of novel therapeutic and diagnostic approaches.

## Teaser

**Background:** *Candida glabrata* virulence is poorly understood at the molecular level.

**Results:** Inactivation of components of the *C. glabrata* glycosylation machinery results in changes in fungal mannan structure and altered virulence.

**Conclusion:** Changes in *C. glabrata* cell wall architecture impact the host-pathogen interactions.

**Significance:** Greater understanding of *C. glabrata* virulence will provide insights that can be adopted for development of novel diagnostic and therapeutic interventions.

## Introduction

The fungal pathogen *Candida glabrata* is a major cause of life-threatening disease in the immunocompromised patient population, causing up to 30% of all candidemias and having a higher attributable mortality than *Candida albicans* (1, 2). As with other pathogenic *Candida* species, the cell wall of *C. glabrata* is the point of contact between host and fungus. In addition, it performs many other functions, protecting the fungal cell from hostile environments, enabling adherence to host surfaces, and maintaining cell shape. The cell walls of *Saccharomyces cerevisiae* and *C. albicans* have both been investigated in detail and extensively reviewed in the literature (3–5). Common to many other fungi, the central core of their cell walls is a branched β-(1,3)-, β-(1,6)-glucan linked to chitin via a β-(1,4)-glucan linkage. This core structure is generally found close to the cell membrane, with chitin innermost, and the β-(1,6)-glucan structure/linkages displayed outwards acting as a linker to the outer cell wall mannoproteins. Some of the chitin and glucan chains extend throughout the entire depth of the cell wall structure (6, 7). The outer glycoprotein layer of the fungal cell wall plays a major role in host recognition (8–15).

These glycoproteins are decorated with both *N-* and *O-*linked sugars, principally mannans, the precise nature of which varies among species but can result in addition of up to 200 mannose units (16). Structural studies indicate that *C. glabrata* mannan is more closely related to that of *S. cerevisiae* than *C. albicans* (17–19), and it shows some inter-strain variation (20). Despite this variation in mannan structure, the core biosynthetic machinery appears to be relatively well conserved, a fact that facilitates the analysis of *C. glabrata* glycosylation. *N*-Linked protein glycosylation occurs in two stages. First, assembly of the core oligosaccharide structure takes place at the membrane of the endoplasmic reticulum (21). The completed core structure is a branched oligosaccharide of residues Glc_3_Man_9_GlcNAc_2_, which is transferred *en bloc* from its lipid anchor to the target asparagine residues on a nascent peptide (22). Once attached, the oligosaccharide is trimmed to leave Man_8_GlcNAc_2_ (23). The second part of the *N*-linked glycosylation process occurs in the Golgi complex, where a single α-(1,6)-linked mannose is added to Man_8_GlcNAc_2_ core by Och1 (24). Proteins either then receive a core-type structure by the addition of two further mannoses or a much more highly decorated α-(1,6)-linked backbone structure, branched by α-(1,2)- and α-(1,3)-mannoses (25, 26). In *S. cerevisiae* this process requires both the *MNN* and *KTR/KRE/MNT* families of mannosyltransferases, including Anp1, Mnn2, and Mnn11 (27–35). It is this final stage in processing that accounts for the huge diversity of glycans decorating fungal glycoproteins. Interestingly, many of these fungal mannosyltransferases are absent from human cells, and hence their analysis has potential with respect to the development of novel antifungal and immunotherapy.

With this in mind, the enzymes involved in both the processing of *N*- and *O*-linked mannans in *C. albicans* have been analyzed and shown to be required for the virulence of this organism, including those specifically involved in both *N*-glycosylation (36) and *O*-glycosylation (37, 38). In addition glycosylation appears to be important in mediating virulence in *Cryptococcus neoformans* (39, 40). Indeed, in *C. neoformans* the polysaccharide capsule that includes mannose-based components is essential for the virulence of this fungus (41). Furthermore, a heterogeneous group of mannoproteins are critical antigens in stimulating T cell responses (42). This importance in virulence may be due in part to differential host recognition. *N*- and *O*-linked mannans are major pathogen-associated molecular patterns and, along with β-glucans, play important roles in triggering host innate immunity. Recent findings in *C. albicans* have highlighted how a coordinated immune response, with stimulus from both *N*- and *O*-linked glycans of the mannoproteins, and also the β-glucan triggers the immune cascade (6). This underlines the fact that multiple components of the cell wall are involved in fungal recognition. Some of the proteins that manufacture these specific epitopes are also required for virulence of *C. albicans* (36, 43, 44).

These studies have resulted in an understanding of how glycosylation of *C. albicans* proteins influences fungal host-pathogen interaction and virulence. However, little is known regarding the role of glycosylation in the pathogenesis of *C. glabrata*. Simultaneous deletion of the *BMT2–6* genes encoding five β-mannosyltransferases yielded a strain that was unable to induce weight loss or chronic inflammation in a murine colitis model (45). Furthermore, nothing is known regarding the conservation of the glycosylation machinery in this species. We therefore sought to determine the effect of inactivation of three putative components of the *C. glabrata N*-linked glycosylation machinery (Anp1, Mnn2, and Mnn11) on cell wall, specifically mannan structure and virulence. We show that there is functional conservation of these enzymes between *C. glabrata* and *S. cerevisiae. ANP1* and *MNN11* encode α-(1–6)-mannosyltransferases, and *MNN2* encodes an α-(1–2)-mannosyltransferase. Inactivation of each gene results in altered *N*-linked mannan structure consistent with these functions. Furthermore, deletion of the genes differentially affects virulence, and this variability may be partially explained by resultant changes in cellular adhesion.

## EXPERIMENTAL PROCEDURES

### 

#### 

##### Strains, Media, and Culture Conditions

All strains used and constructed in this study are listed in Table 1. Fungal cells were routinely cultured in yeast extract peptone dextrose (YPD) (2% (w/v) peptone, 2% (w/v) glucose, 1% (w/v) yeast extract), yeast extract peptone maltose (YPM) (2% (w/v) peptone, 2% (w/v) maltose, 1% (w/v) yeast extract), or synthetic dropout medium (SD) (0.68% (w/v) yeast nitrogen base without amino acids (Difco), 2% (w/v) glucose, and appropriate dropout mix (Clontech) at 30 °C (*S. cerevisiae*) or 37 °C (*C. glabrata*) at 180 rpm. For culture on solid media 2% (w/v), agar was added prior to autoclaving. For phenotypic assays, selective media were made as described by Hampsey (46). Strains were stored at room temperature for up to 4 weeks on solid agar plates or for long term storage in 50% (v/v) glycerol at −80 °C.

**TABLE 1:** **Fungal strains used in this study**

Species	Strain	Genotype or description	Source
*C. glabrata*	ATCC 2001	Wild type	ATCC
*C. glabrata*	ΔHT6	Δ*his3*::Sc*URA3* Δ*trp1*	48
*C. glabrata*	XFS-1	Δ*his3*::*ScURA3* Δ*trp1* Δ*anp1*::*HIS3*	This study
*C. glabrata*	XFS-1P	Δ*his3*::*ScURA3* Δ*trp1* Δ*anp1*::*HIS3* pCgACT14 (*TRP1*)	This study
*C. glabrata*	LJW-5RLP	Δ*his3*::*URA3* Δ*trp1* Δ*anp1*::*ANP1* pCgACT14 (*TRP1*) pCgACH3 (*HIS3*)	This study
*C. glabrata*	LJW-2	Δ*his3*::*URA3* Δ*trp1* Δ*mnn2*::*HIS3*	This study
*C. glabrata*	LJW-2P	Δ*his3*::*URA3* Δ*trp1* Δ*mnn2*::*HIS3* pCgACT14 (*TRP1*)	This study
*C. glabrata*	LJW-2RLP	Δ*his3*::*URA3* Δ*trp1* Δ*mnn2*::*MNN2* pCgACT14 (*TRP1*) pCgACH3 (*HIS3*)	This study
*C. glabrata*	LJW-3	Δ*his3*::*URA3* Δ*trp1* Δ*mnn11*::*HIS3*	This study
*C. glabrata*	LJW-3P	Δ*his3*::*URA3* Δ*trp1* Δ*mnn11*::*HIS3* pCgACT14 (*TRP1*)	This study
*S. cerevisiae*	BY4741	*MAT***a** Δ*his3* Δ*leu2* Δ*met15* Δ*ura3*	51
*C. glabrata*	LJW-6	Δ*his3*::*URA3* Δ*trp1* Δ*anp1*::*HIS3* pLJW5 (*TRP1 ScANP1*)	This study
*S. cerevisiae*	LJW-8	*MAT***a** *mnn2*Δ::*kanMX4* Δ*his3* Δl*eu2* Δ*met15* Δ*ura3* pLJW7 (*LEU2 CgMNN2*)	This study
*S. cerevisiae*	L9	*MAT***a** *mnn11*Δ::*kanMX4* Δ*his3* Δl*eu2* Δ*met15* Δ*ura3* pLJW8 (*LEU2 CgMNN11*)	This study

##### Construction of C. glabrata Mutants

To disrupt *C. glabrata* genes, a one-step PCR-based approach was adopted (47). DNA fragments were amplified using primer pairs such that the PCR product would contain 60 bp of homology to the gene of interest at both the 5′ and 3′ ends and 20-bp tails homologous to the *C. glabrata HIS3* gene, which was amplified from pTW25 (48). Primer sequences are available upon request. The disruption cassette was transformed into *C. glabrata* ΔHT6, and histidine prototrophs were selected on appropriate dropout media. To reconstitute *C. glabrata* genes of interest, the *SAT1* flipper method was used (49). Southern analysis was used to confirm gene disruption at the correct locus and single integration.

The *C. glabrata anp1* null mutant was constructed by removing 1344 bp of the *C. glabrata ANP1* gene (CAGL0L01331g, +1 to +1344 with respect to the start codon, and the stop codon is at +1342) via homologous recombination. Four independent transformants were selected. These strains were all screened in a full phenotypic assay (data not shown), and one mutant was selected for further study, *C. glabrata* XFS-1 (*anp1*). This was made prototrophic by transformation with pCgACT14 (50) to give *C. glabrata* XFS-1P.

To reconstitute *ANP1* in *C. glabrata* XFS-1 plasmids, pLJW6 and pLJW7 were constructed as follows. A NotI-SacII downstream fragment of the *C. glabrata ANP1* gene (positions +1322 to +1772) was amplified from *C. glabrata* 2001 genomic DNA. The resulting 459-bp downstream fragment was digested with NotI and SacII and cloned into NotI-SacII-digested pSFS2 (49) to generate plasmid pLJW6. A KpnI-XhoI fragment containing the complete open reading frame as well as 0.44 kb of upstream and 0.44 kb of downstream flanking sequences of the *ANP1* gene was amplified from *C. glabrata* 2001 genomic DNA. The resulting 2164-bp fragment was digested with KpnI and XhoI and cloned into KpnI-XhoI digested pLJW6 to generate pLJW7. The insert from plasmid pLJW7 was excised as a KpnI-SacII fragment for transformation into *C. glabrata* XFS-1 by electroporation. Cells were spread on YPD plates containing 200 μg/ml nourseothricin and cultured at 37 °C for 96 h. Four independent transformants were inoculated into YPM liquid medium overnight without nourseothricin to allow for *FLP*-mediated excision of the *SAT1* flipper and nourseothricin-sensitive strains selected on YPD plates containing 10 μg/ml nourseothricin as detected by their smaller colony size compared with nourseothricin-resistant strains. Southern analysis was used to confirm flipper excision, gene integration at the correct locus, and single integration. Strains were made fully prototrophic by transformation with pCgACT14 and pCgACH3 (50), and then each transformant was subjected to a phenotypic screen (data not shown), and a single strain, *C. glabrata* LJW-5RLP (*anp1*::*CgANP1*), was selected for further study.

The *C. glabrata mnn2* null mutant was constructed by removing 1839 bp of the *C. glabrata MNN2* gene (CAGL0I04532g, +1 to +1839 with respect to the start codon; the stop codon is at +1837) via homologous recombination. Four independent transformants were selected. These strains were all screened in a full phenotypic assay (data not shown), and one mutant, *C. glabrata* LJW-2 (*mnn2*), was selected for further study. This was made prototrophic by transformation with pCgACT14 to give *C. glabrata* LJW-2P.

To reconstitute *MNN2* in *C. glabrata,* LJW-2 plasmids pLJW8 and pLJW9 were constructed as follows. A NotI-SacII downstream fragment of the *C. glabrata MNN2* gene (positions +1820 to + 2250) was amplified from *C. glabrata* 2001 genomic DNA. The resulting 331-bp downstream fragment was digested with NotI and SacII and cloned into NotI-SacII digested pSFS2 to generate plasmid pLJW8. An ApaI-XhoI fragment containing the complete open reading frame as well as 0.44 kb of upstream and 0.30 kb of downstream flanking sequences of the *MNN2* gene were amplified from *C. glabrata* 2001 genomic DNA. The resulting 2583-bp fragment was digested with ApaI and XhoI and cloned into ApaI-XhoI-digested pLJW8 to generate pLJW9. The insert from plasmid pLJW9 was excised as an ApaI-SacII fragment for transformation into *C. glabrata* LJW-2 by electroporation, and re-integrants were selected, made prototrophic, and confirmed as above to yield *C. glabrata* LJW-2RLP (*mnn2*::*CgMNN2*).

The *C. glabrata mnn11* null mutant was constructed by removing 1326 bp of the *C. glabrata MNN11* gene (CAGL0G07491g, +1 to +1326 with respect to the start codon, the stop codon is at +1324) via homologous recombination. Four independent transformants were selected. These strains were all screened in a full phenotypic assay (data not shown), and one mutant, *C. glabrata* LJW-3 (*mnn11*), was selected for further study. This was made prototrophic by transformation with pCgACT14 to give *C. glabrata* LJW-3P. *MNN11* was not reconstituted.

##### Cross-species Complementation

To determine whether the functions encoded by the *C. glabrata* and *S. cerevisiae ANP1*, *MNN2,* and *MNN11* orthologues have been conserved, we performed a series of cross-species complementation experiments. First, we sought to determine whether *S. cerevisiae ANP1* could complement phenotypes of the *C. glabrata anp1* mutant. To achieve this, the entire *S. cerevisiae ANP1* open reading frame (−760 to +2480, the stop codon is at +1501) was amplified from *S. cerevisiae* BY4741 (51) genomic DNA. The resulting 3287-bp product was cloned directly into pGEM-T Easy (Promega), excised with BamHI, and cloned into BamHI-digested pCgACT14 to give plasmid pLJW1. pLJW1 was transformed into *C. glabrata* XFS-1 (*anp1*), and tryptophan prototrophs were selected. A representative strain was selected and designated *C. glabrata* LJW-6 (*anp1*::*ScANP1*).

Next, we sought to determine whether *C. glabrata MNN2* and *MNN11* could complement the phenotypes of the *S. cerevisiae mnn2* and *mnn11* mutants, respectively. To achieve this, the entire *C. glabrata MNN2* (−1265 to +2376, the stop codon is at +1837) and *MNN11* (−1916 to +1911, the stop codon is at +1324) open reading frames plus flanking regions were amplified from *C. glabrata* 2001 genomic DNA. The resulting 3661- and 3779-bp products were cloned directly into pGEM-T Easy, excised with BamHI, and cloned into BamHI-digested YCp111 (52) to give plasmids pLJW3 and pLJW4, respectively. pLJW3 was transformed into *S. cerevisiae mnn2,* and leucine prototrophs were selected. A representative strain was selected and termed *S. cerevisiae* LJW-8 (*mnn2*::*CgMNN2*). pLJW4 was transformed into *S. cerevisiae mnn11,* and leucine prototrophs were selected. A representative strain was selected and termed *S. cerevisiae* LJW-9 (*mnn11*::*CgMNN11*).

##### Virulence Analysis

We then sought to determine how inactivation of *ANP1*, *MNN2*, and *MNN11* impacted the ability of *C. glabrata* to cause disease in a well established murine model of systemic candidosis. To achieve this virulence, analysis was performed essentially as described previously (53–55). Briefly, groups of 10–22 out-bred male CD1 mice were immunosuppressed with 200 mg of cyclophosphamide/kg of body weight on day −3 and every 4th day thereafter. Animals were infected with 7 × 10^7^ *C. glabrata* yeast cells in 200 μl of saline via tail vein injection. Following infection, mice were weighed and observed daily and sacrificed at predetermined end points, *e.g.* 20% weight loss.

##### Ethics Statement

All animal work was performed under the auspices of the “Animals (Scientific Procedures) Act 1986” at Imperial College London, United Kingdom. All protocols were approved by the Home Office under project license PPL 70/6487.

##### Alcian blue Binding Assay

Alcian blue binding assays were performed essentially as described previously to determine the extent of mannan phosphorylation (58). Briefly, a suspension of 1 × 10^7^ washed exponential phase cells was suspended in 1 ml of 30 μg/ml Alcian blue in 0.02 m HCl (pH 3), incubated at room temperature for 10 min, and pelleted by centrifugation. Then *A*_600_ values of 100 μl of supernatant samples were determined in a spectrophotometer. Alcian blue concentration was determined by reference to a standard curve (microgram of Alcian blue bound per *A*_600_ unit of cell suspension).

##### Mannan Isolation

To analyze the consequences of gene deletion on mannan structure, mannan was isolated using a modified method first described by Kocourek and Ballou (59). Briefly, 1 liter of saturated culture was collected by centrifugation, and the cells were washed in double distilled water. Washed cells were resuspended in an excess of acetone; the cells were collected by centrifugation, and the supernatant acetone was removed. The cells were dried over Drierite® and under vacuum. The cells were rehydrated in 200 ml of double distilled water and subjected to autoclaving for 3 h, and after cooling the solid extract was collected by centrifugation and the remaining supernatant subjected to Fehling precipitation. An equal volume of Fehling's solution (50:50 Fehling's Solution No. 1 and No. 2) was added to the extracted mannan mixture with stirring, and a precipitate of copper-mannan was then formed and allowed to settle. The remaining supernatant was removed, and the copper complex was dissolved in 6 ml of 3 m HCl. The resulting solution was poured slowly, with stirring, into a 100-ml (8:1) mixture of methanol/acetic acid, and the resulting precipitate was allowed to settle overnight. The supernatant was decanted, and the precipitate was stirred with a fresh methanol/acetic acid mixture to remove the copper complex. This was repeated until the solution appeared colorless. The precipitate was collected and washed several times with methanol and allowed to dry under vacuum.

##### Proton and Carbon-13 NMR

Structural analysis of the mannan extracts was performed using one- and two-dimensional proton (60) and carbon-13 NMR (61). NMR spectra were collected on a JEOL Eclipse+ 600 NMR spectrometer operating at 80 ± 1 °C in 5-mm NMR tubes. Mannan was dissolved in D_2_O at 80 ± 1 °C. Proton chemical shifts were referenced to sodium 3-trimethylsilylpropionate-2,2,3,3-*d*_4_. C-13 chemical shifts were referenced to external acetone. Proton one-dimensional NMR spectral collection and processing parameters were as follows: 25 ppm spectral width centered at 7.5 ppm, 32,768 data points, 1024 scans, 15 s relaxation delay, 2.18 s acquisition time, and exponential apodization. C-13 one-dimensional NMR spectral collection and processing parameters were as follows: 250 ppm spectral width centered at 110 ppm, 65,536 data points, 3161 scans, 5 s relaxation delay, 1.74 s acquisition time, and exponential apodization. Homonuclear gradient COSY two-dimensional NMR spectra were collected and processed as follows: 512 × 128 point matrix was zero-filled to 512 × 1024 points, 256 scans per row with 4 dummy scans, 3 ppm sweep width centered at 4.5 ppm, sinebell apodization in both dimensions, and 1 s relaxation delay. NMR spectra were processed using JEOL DELTA software running on the Eclipse+ 600 NMR and on a Macintosh MacBook Pro.

##### Gel Permeation Chromatography-Multiangle Laser Light Scattering (GPC/MALLS)^3^ Detection

Further structural analysis was performed by high performance GPC/MALLS photometry as reported previously by Müller *et al.* (62) and Adams *et al.* (63) to determine the polysaccharide weight averaged molecular mass and root mean square(r.m.s.) radius. Briefly, the mannan samples were dissolved at 3 mg/ml, heated for 15 min at 60 °C, cooled, and filter-sterilized in 50 mm sodium nitrite mobile phase. Three Ultrahydrogel columns (1200, 500, and 100; Waters) were connected in a series, and the columns were maintained at 37 ± 1 °C with continuous mobile phase flow. The system was calibrated using narrow band pullulan standards (Showa Denko, Japan). The weight-average molecular mass and the *z* average radius of the center of gravity as an index of molecular size of the samples were determined by on-line MALLS photometry employing a Wyatt Technology TriStar MALLS (λ = 690 nm) photometer. Data were acquired and analyzed using Astra software (version 4.9; Wyatt Technology).

##### Flow Adhesion Assay of C. glabrata Mutants

To determine whether gene deletion affected cellular adherence, we used a well established endothelial flow assay performed essentially as described previously (64). Briefly, *C. glabrata* was cultured overnight in liquid YPD at 37 °C, 180 rpm, washed three times with sterile Hanks' buffered salt solution (Invitrogen), counted, and resuspended at 1.0 × 10^6^ yeast/ml in Hanks' buffered salt solution. Glass slides coated with confluent HMEC-1 endothelial cell monolayers were mounted in a parallel plate flow chamber (GlycoTech, Rockville, MD), and *C. glabrata* cells were perfused through the flow chamber and over the endothelial cell monolayer, using an automated syringe pump at 0.25 dynes/cm^2^ (Harvard Apparatus, Natick, MA). All experiments were performed on a 37 °C stage, in an environmental microscope chamber also maintained at 37 °C. Adhesion events were visualized using a Zeiss Axiovert 200 M inverted fluorescence microscope. An integrated high resolution AxioCam digital camera (Nikon) with Axiovision 4.6 software (Imaging Associates Ltd., Bicester, UK) was used to record the flow experiments. *C. glabrata* suspensions were allowed to perfuse the flow chamber for 2 min before commencing recording. Results consisted of 15-min recordings of a random field of view (0.15 mm^2^) using a ×20 objective. Each experiment was repeated with three separate confluent endothelial cell slides on at least two occasions. Cell motion analysis was performed using time-lapse software. Images were then acquired over 15 min into a video file at 2 frames/min, and the total number of adherent cells/mm^2^ was recorded.

## RESULTS

### 

#### 

##### ANP1, MNN2, and MNN11 Gene Functions Are Conserved between C. glabrata and S. cerevisiae

**FIGURE 1.**
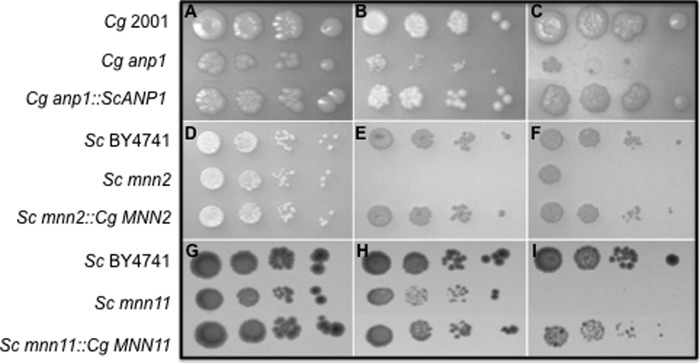
***C. glabrata* 2001 *ANP1, MNN2,* and *MNN11* encode functional homologues of *S. cerevisiae* Anp1, Mnn2, and Mnn11.** *A–I,* 10-fold serial dilution of yeast strains were cultured on YPD (*A, D,* and *G*); YPD plus 10 mm caffeine (*B*); YPD plus 1 mg/ml Calcofluor White (*C* and *F*); YPD plus 50 μg/ml hygromycin (*I*), or YPD plus 3 mm sodium orthovanadate (*E* and *H*) at 37 °C (*A–C*) or 30 °C (*D–I*) for 48 h. *S. cerevisiae ANP1* complements the growth defects of *C. glabrata anp1* cells on caffeine (*B*) and Calcofluor White (*C*). Similarly, *C. glabrata MNN2* and *MNN11* complement the growth defects of *S. cerevisiae mnn2* and *mnn11* cells, respectively on sodium orthovanadate (*E* and *H*); Calcofluor White (*F*), and hygromycin (*I*). This demonstrates that *C. glabrata* and *S. cerevisiae ANP1, MNN2* and *MNN11* encode at least partial functional homologues.

To determine whether *C. glabrata ANP1, MNN2,* and *MNN11* encode functional homologues of the *S. cerevisiae* α-(1–6)-mannosyltransferases (Anp1 and Mnn11) and α-(1–2)-mannosyltransferase (Mnn2), we conducted a series of cross-complementation experiments. *S. cerevisiae ANP1* was able to successfully rescue the caffeine, SDS, Calcofluor White, hygromycin B, and NaCl sensitivities of the *C. glabrata anp1* null mutant (Fig. 1, *A–C*, and data not shown). Similarly *C. glabrata MNN2* and *MNN11* were able to rescue phenotypes associated with *S. cerevisiae mnn2* and *mnn11* null mutants, respectively (Fig. 1, *D–I*, and data not shown). This demonstrates that the genes in the two species encode at least partial functional homologues.

**FIGURE 2.**
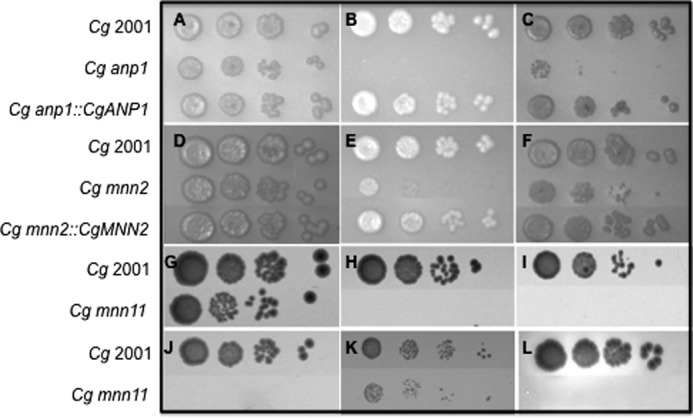
***C. glabrata anp1* and *mnn2* cells exhibit phenotypes associated with weakened cell walls.** *A–L,* 10-fold serial dilution of yeast strains were cultured on YPD (*A, D,* and *G*); YPD plus 1 mg/ml Calcofluor White (*B, E,* and *L*); YPD plus 50 μg/ml hygromycin (*C, F* and *J*); YPD plus 3 mm sodium orthovanadate (*I*); YPD plus 1.5 m NaCl at 37 °C for 48 h (*K*), or YPD at 42 °C for 48 h (*H*). *C. glabrata anp1* (*A–C*) and *mnn2* mutants (*D–F*) exhibit reduced growth, compared with wild-type *C. glabrata* cells, on Calcofluor White (*B* and *E*) and hygromycin (*C* and *F*). Reintegration of *C. glabrata ANP1* (*A–C*) or *MNN2* (*D–F*) into the appropriate mutant background restored wild-type growth characteristics. *H–L*, *C. glabrata mnn11* mutants exhibit reduced growth, compared with wild-type *C. glabrata* cells, *at 42* °*C (H*) and in the presence of sodium orthovanadate (*I*), hygromycin (*J*), NaCl (*K*), and Calcofluor White (*L*).

In addition to these complementation studies, we determined the consequences of the individual *C. glabrata* gene deletions to various perturbations. As anticipated, all three mutants had phenotypes consistent with a weakened cell wall. The *C. glabrata anp1* null mutant was hypersensitive to the cell wall perturbing agents Calcofluor White and SDS and the stress-inducing agent NaCl. Furthermore, analogous to glycosylation-defective strains in *S. cerevisiae,* the *C. glabrata anp1* null mutant was hypersensitive to hygromycin B (Fig. 2, *A–C*, data not shown). Comparatively, *C. glabrata mnn2* was unaffected for growth on hygromycin B, NaCl, and SDS but harbored a cell wall defect of sorts as the null mutant was hypersensitive to Calcofluor White and sodium orthovanadate (Fig. 2, *D–F*, data not shown). *C. glabrata mnn11* was hypersensitive to Calcofluor White, NaCl, hygromycin B, sodium orthovanadate, and to growth at 42 °C (Fig. 2, *G–L*). In liquid culture, all three null mutants exhibited slight growth defects with doubling times of 54 min (*mnn11*), 60 min (*mnn2*), and 70 min (*anp1*) in YPD at 37 °C compared with 45 min for *C. glabrata* 2001. In addition, *C. glabrata mnn2* and *mnn11* tended to form small cellular aggregates that could be largely dispersed by vigorous vortexing. These data strongly support the hypothesis that *C. glabrata* Anp1, Mnn2, and Mnn11, as expected for mannosyltransferases, play roles, as do their counterparts in *S. cerevisiae*, in maintaining cell wall integrity.

##### Structure of C. glabrata Mannan

Our cross-complementation experiments strongly suggest that *C. glabrata ANP1, MNN2,* and *MNN11* encode functional homologues of the respective *S. cerevisiae* mannosyltransferases. Hence, we would anticipate that their inactivation should result in changes to mannan structure, specifically that the *anp1* and *mnn11* mutants would have shorter α-(1–6)-polymannosyl backbones, as they would have reduced α-(1–6)-mannosyltransferase activity, and the *mnn2* null would lack α-(1–2)-mannose side chains, due to loss of α-(1–2)-mannosyltransferase. To verify this, we carried out a series of physicochemical and structural analyses.

**FIGURE 3.**
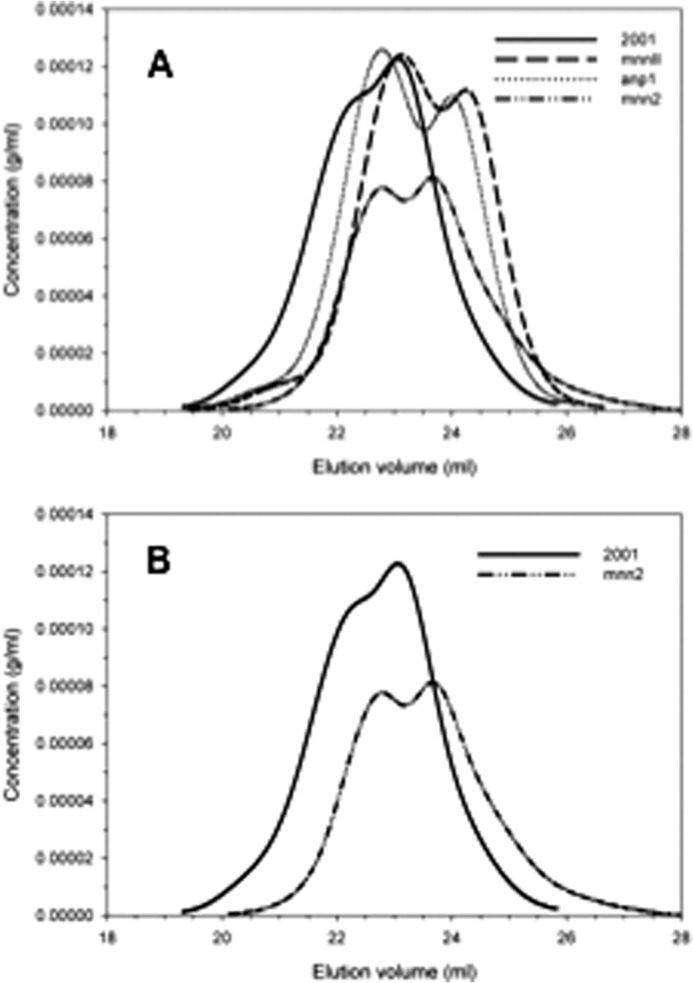
**Molecular weights of *C. glabrata anp1, mnn2,* and *mnn11* mannans are lower than mannan isolated from wild-type cells.** *A,* refractive index (concentration) chromatogram of *C. glabrata anp1* (*dotted line*), *mnn2* (*hashed* and *dotted line*), and *mnn11* (*hashed line*) mannans are shifted downfield indicating a lower molecular weight than mannan isolated from wild-type (*solid line*) cells. *B,* this is particularly evident in the downfield shift for the *mnn2* mannan (*hashed* and *dotted line*) when compared with wild-type mannan (*solid line*). The average molecular weight, polydispersity, and r.m.s. radii for all four mannans and a pullulan standard are shown in detail in Table 2. All mannan samples were passed through three Ultrahydrogel columns (1200, 500, and 100, Waters) connected in series, and the weight-average molecular mass, polydispersity, and r.m.s. radius of the samples were determined by on-line MALLS photometry as described under “Experimental Procedures.”

Initially, GPC/MALLS was used to compare the molecular weight and r.m.s. radii of mannans from *C. glabrata* 2001, *anp1*, *mnn2*, and *mnn11* strains. Pullulan was used as a control. The mannans from all three mutants showed polymer distributions that were different from *C. glabrata* 2001. Specifically, mannans from the mutants were characterized by a larger quantity of lower molecular weight polymers (Fig. 3*A* and Table 2). For example, the polymer distribution of *C. glabrata mnn2* mannan is clearly shifted downfield in the GPC/MALLS chromatogram (Fig. 3*B*) which is indicative of a lower molecular weight mannan compared with the wild-type *C. glabrata* 2001 strain. This was also the case for mannans from *anp1* and *mnn11* cells, as would be expected if the mannan polymers had a lower degree of polymerization as a result of a shorter α-(1–6) backbone or missing α-(1–2) side chains. The r.m.s. radius provides an indication of the volume that the molecules occupy in three-dimensional space. *C. glabrata mnn2* mannan has a 47.4% reduction in r.m.s. radius compared with *C. glabrata* 2001, suggesting that it contains the fewest mannose monosaccharides of all strains tested. Reductions of 29.2 and 29.9% were observed in the *anp1* and *mnn11* mannans (Table 2).

**TABLE 2:** **Comparison of molecular weight and rms radii for mannans isolated from *C. glabrata* 2001, *anp1*, *mnn2* and *mnn11***

Strain	Molecular weight*^a^*	r.m.s. radius*^b^*
	*g/mol*	*nm*
Pullulan*^c^*	1.10 × 10^5^	25.4
2001	1.54 × 10^5^	25.4
*anp1*	1.09 × 10^5^	22.1
*mnn2*	0.81 × 10^5^	26.1
*mnn11*	1.08 × 10^5^	23.6

*^a^* Molecular weight is expressed as weight average molecular weight in g/mol. It is representative of the average molecular weight over the entire polymer distribution.

*^b^* The r.m.s. radius is a measure of a polymer's size weighted by the mass distribution about its centre of mass.

*^c^* Pullulan (Showa Denko, Japan) was used as a standard.

**FIGURE 4.**
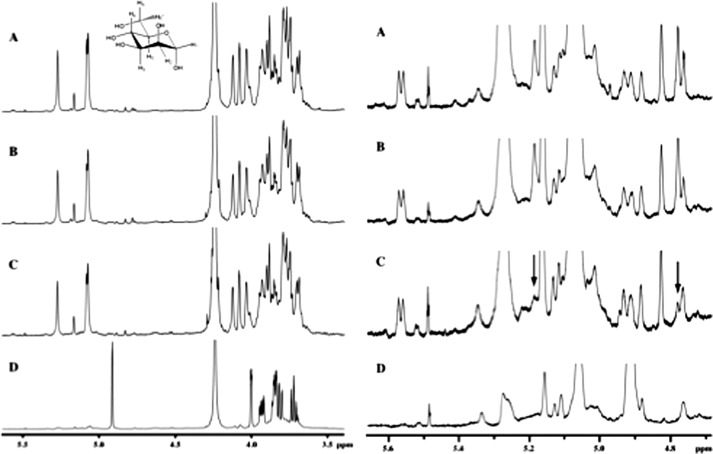
***C. glabrata mnn2* mannan is distinct from other mannans analyzed and lacks α-(1,2)-linked mannosyl side chains, whereas *mnn11* mannan is similar to 2001 and *anp1* mannans but contains a reduced level of Mβ1–2Mα1 side chains.** Comparison of mannan ^1^H NMR spectra for *C. glabrata* 2001 (*A*), *anp1* (*B*), *mnn11* (*C*), and *mnn2* (*D*). The carbohydrate spectral regions for all four mannans plotted from 3.4 to 5.6 ppm are compared on the *left*, and the expanded anomeric proton spectral regions for all four mannans plotted from 4.7 to 5.65 ppm are compared on the *right*. The mannan from *C. glabrata mnn2* is distinctly different from the other mannans because it lacks resonances in the 5.0–5.3 ppm spectral region for α-(1,2)-linked mannosyl side chain repeat units. The mannan from *mnn11* is similar to but slightly different compared with mannans from 2001 and *anp1* due to the reduced level of Mβ1–2Mα1 end groups indicated by the smaller resonances near 4.78 and 5.18 ppm (indicated by *vertical arrows*). Proton NMR spectra were collected on a JEOL Eclipse+ 600 NMR spectrometer operating at 80 ± 1 °C in 5-mm NMR tubes. Each mannan isolate was dissolved in D_2_O at 80 °C ± 1 °C. Proton chemical shifts were referenced to sodium 3-trimethylsilylpropionate-2,2,3,3-*d*_4_.

**FIGURE 5.**
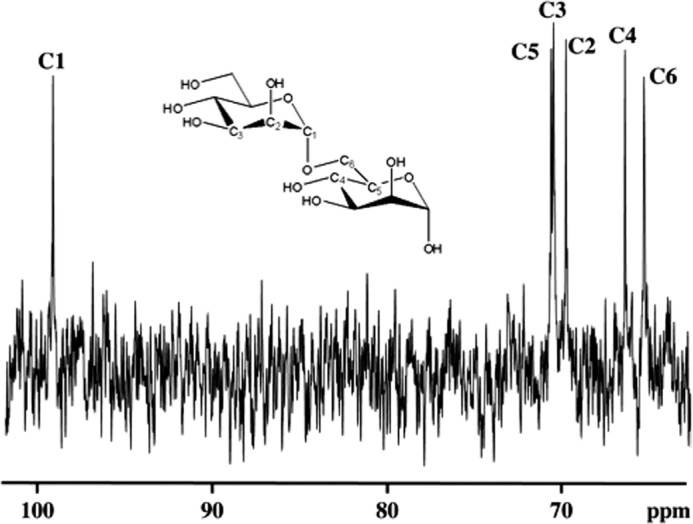
***C. glabrata mnn2* cell wall contains α-(1,6)-mannan.** The ^13^C NMR spectrum of α-(1,6)-mannan extracted from *C. glabrata mnn2* cells is shown. Resonance chemical shifts are assigned to specific carbons in Table 4. Resonances are labeled and assigned based on the α-(1,6)-linked mannan dimer shown (61).

To further characterize these differences we performed ^1^H NMR studies. Chemical shift assignments for the anomeric proton, H1, and H2 of the mannosyl repeat units in the backbone and side chain structural fragments were obtained from COSY spectra (data not shown). These studies show (Table 3) that *C. glabrata* 2001, *anp1,* and *mnn11* mannans (Fig. 4, *A–C*, respectively) are distinctly different from the mannan isolated from *C. glabrata* mnn2 (Fig. 4*D*). Specifically, the *C. glabrata* 2001, *anp1*, and *mnn11* mannans exhibit resonances that are assigned to structural fragments in α-(1,2)-linked (60) and possibly α-(1,3)-linked (65) mannosyl repeat units in side chains attached to the α-(1,6)-linked backbone chain with all backbone repeat units containing side chains (Table 3). Also, the presence of mannosyl repeat units associated with the phosphodiester linkage between acid-stable and acid-labile portions of the mannan structure is evident for 2001, *anp1*, and *mnn11*. In addition, the proton NMR spectrum of *mnn11* mannan (Fig. 4*C*) indicates a reduced level of Mβ1–2Mα1, side chains compared with 2001 and *anp1* mannans based on the reduced intensity of the resonances near 4.78 and 5.18 ppm (Fig. 4*C*, indicated by *vertical arrows*). These resonances are assigned to the anomeric protons of the Mβ1- and -2Mα1 structural fragments, respectively, of the Mβ1–2Mα1 side chain structural fragment. The predominant resonances for *C. glabrata mnn2* mannan support the presence of backbone mannosyl repeat units without attached side chains based upon the major anomeric proton resonance at 4.91 ppm. This singlet resonance at 4.91 ppm is assigned to an anomeric proton of the α-(1,6)-linked backbone mannosyl repeat unit (-Mα1–6Mα1–6Mα-) containing no side chains (66, 67). Also, significant side chain structural fragments containing α-(1,2)-linked and possibly α-(1,3)-linked mannosyl repeat units (60, 65) are not indicated based upon the reduced level of these resonances in the 5.0–5.3 ppm spectral region. The side chains in the *mnn2* mannan are considerably shorter than in 2001, *anp1*, and *mnn11* mannans. Predominant side chain structure arises from side chains containing single α-(1,2)- or α-(1,3)-linked mannosyl repeat units or short chains containing single α-(1,2)- or α-(1,3)-linked mannosyl repeat units terminated by 1–3 β-(1,2)-linked mannosyl repeat units. In addition, only the acid-stable portion of the mannan structure is observed in the *mnn2* mannan because anomeric protons associated with acid-labile mannosyl repeat units in side chains attached to a phosphodiester linkage are not evident (Table 3). ^13^C NMR (Fig. 5) with six resonances whose chemical shifts compare favorably with an α-(1,6)-mannan dimer model compound (61) confirms this structural assignment. Proton and ^13^C NMR chemical shift assignments along with structural fragment and dimer chemical shifts for *C. glabrata mnn2* mannan are summarized in Table 4. Therefore, *C. glabrata mnn2* expresses nearly pure α-(1,6)-mannan with fewer or shorter side chains, whereas *C. glabrata* 2001, *anp1*, and *mnn11* express mannan containing side chains (Table 3).

**TABLE 3:** **Significant mannosyl structural fragments present in the mannans purified from *C. glabrata 2001, anp1, mnn2, and mnn11***

Structural fragments*^a^*	2001	*anp1*	*mnn11*	*mnn2*
→2Μα1→PO_4_	Yes	Yes	Yes	No
α1→2 mα1→2	Yes	Yes	Yes	Yes
Mβ1–2 mα1–2(3)*^b^*	Yes	Yes	Minor	No
Μα1→2(3)	Yes	Yes	Yes	Yes
Μα1→3	Yes	Yes	Yes	Yes
1→6 m(→2)1→6 m(→2 mα1)α1→6 m(→2)1→	Yes	Yes	Yes	Yes
1→6 m(→2)1→6 m(→2 mα1→2 mα1)α1→6 m(→2)1→	Yes	Yes	Yes	No
α1→3 mα1→2	Yes	Yes	Yes	No
→6 m(→2 mα1)α1→6 m(→2 mα1→2)α1→6	No	No	No	Yes
α1→2 mα1→2	No	No	No	Yes
α1→6 mα1→6	No	No	No	Yes
Mβ1(→2Μβ1)_n_→2Μα1→(2(3) or PO_4_) [*n* = 1 or 2]	Yes	Yes	Yes	Yes
Mβ1→2Μα1→PO_4_	Yes	Yes	Yes	No
Mβ1→2Μα1→2	Yes	Yes	Minor	No
Mβ1→2Μα1→	No	No	No	Yes

*^a^* Structural fragment identifications are based upon analysis of COSY two-dimensional NMR spectra observed for each isolate. In each fragment, “M” refers to the mannosyl repeat unit. The number represents the point of attachment within the specified mannosyl repeat unit. Proton numbering is indicated in the monomer structure shown in Fig. 4. α and β indicate the conformation at the anomeric carbon.

*^b^* “2(3)” indicates that the point of attachment to the neighboring mannosyl repeat unit could be either at the 2- or 3-position.

**TABLE 4:** ***C. glabrata mnn2* mannan ^1^H and ^13^C NMR chemical shifts** *C. glabrata mnn2* mannan ^1^H and ^13^C NMR chemical shifts (in ppm) are compared with the reference assignments for protons, and an α-(1,6)-mannan dimer (61) for ^13^C. Proton labels are shown in the mannose monomer structure in Fig. 4. The structure of the dimer with carbon labels is shown in Fig. 5. Proton and ^13^C NMR spectra were collected on a JEOL Eclipse+ 600 NMR spectrometer operating at 80 °C ± 1 °C in 5-mm NMR tubes. Mannan was dissolved in D_2_O at 80 °C ± 1 °C. Proton chemical shifts were referenced to sodium 3-trimethylsilylpropionate-2,2,3,3-*d*_4_. C-13 chemical shifts were referenced to external acetone.

Nucleus	^1^H NMR		^13^C NMR	
	This work	Reference assignment	This work	Dimer (61)
1	4.911	4.920 (66) 4.918 (60)	99.38	100.3
2	4.002	4.009 (66) 4.011 (60)	70.02	70.8
3	3.854	3.856 (60)	70.72	71.5
4	3.736		66.64	67.4
5	3.854		70.86	71.6
6	3.943		65.56	66.5
6′	3.812			

**FIGURE 6.**
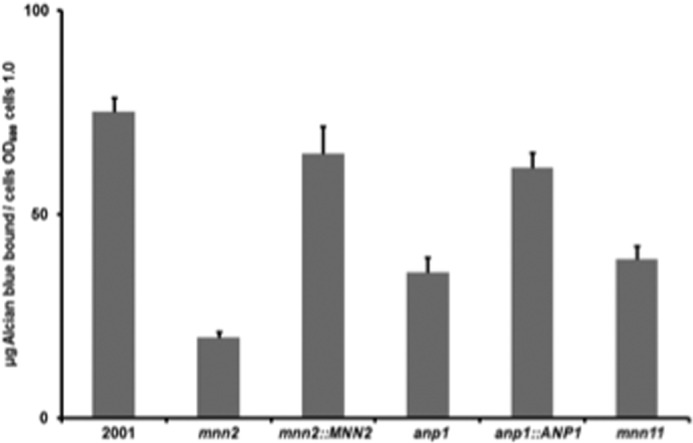
***C. glabrata anp1, mnn2,* and *mnn11* cells are deficient in mannosyl phosphorylation.** Binding of Alcian blue to *C. glabrata* 2001, *anp1*, *anp1*::*ANP1*, *mnn2*, *mnn2*::*MNN2,* and *mnn11 cells. C. glabrata anp1*, *mnn2,* and *mnn11* cells show statistically significant reduced levels of Alcian blue binding compared with wild-type cells (*p* < 0.05, *t* test). Reintegration of *MNN2* to the appropriate null mutant restored the capacity to bind Alcian blue (*p* > 0.05, *t* test), and reintegration of *ANP1* partially restored Alcian blue binding capacity. Results (mean ± S.D.) are pooled triplicate data from a representative experiment. Exponentially growing *C. glabrata* cells were suspended in Alcian blue, incubated at room temperature for 10 min, and pelleted by centrifugation. Then, *A*_600_ values of 100 μl supernatant samples were determined in a spectrophotometer, and Alcian blue concentration was determined by reference to a standard curve.

The Alcian blue assay showed that inactivation of *MNN2* resulted in a 75% reduction in mannosyl phosphorylation compared with wild-type cells, whereas the relative reduction was only 50% when *ANP1* or *MNN11* was deleted (Fig. 6). Furthermore, the *C. glabrata* 2001 ^1^H NMR spectrum shows a doublet resonance at 5.57 ppm from phosphorylation of the mannan (Fig. 4*B*), which the *C. glabrata mnn2* spectrum lacks (Fig. 4*D*). This supports the data from the Alcian blue experiments of decreased mannosyl phosphorylation in the *mnn2* null mutant, and it suggests that the α-(1,2)-mannose side branches are important sites for mannosyl phosphate linkage.

Taken together, these data strongly support the view that Anp1, Mnn2, and Mnn11 function as mannosyltransferases in *C. glabrata*, and both *S. cerevisiae* and *C. glabrata* mannans are structurally similar but distinct from *C. albicans* mannan (65).

##### Role of C. glabrata Anp1, Mnn2, and Mnn11 in Virulence

Having determined that *C. glabrata ANP1, MNN2,* and *MNN11* encode functional homologues of the orthologous *S. cerevisiae* proteins, we sought to investigate their role in virulence. To achieve this, we used an established murine model of systemic infection (53–56). Groups of neutropenic male CD1 mice were inoculated with 7 × 10^7^ *C. glabrata* cells and were followed as described under “Experimental Procedures.”

**FIGURE 7.**
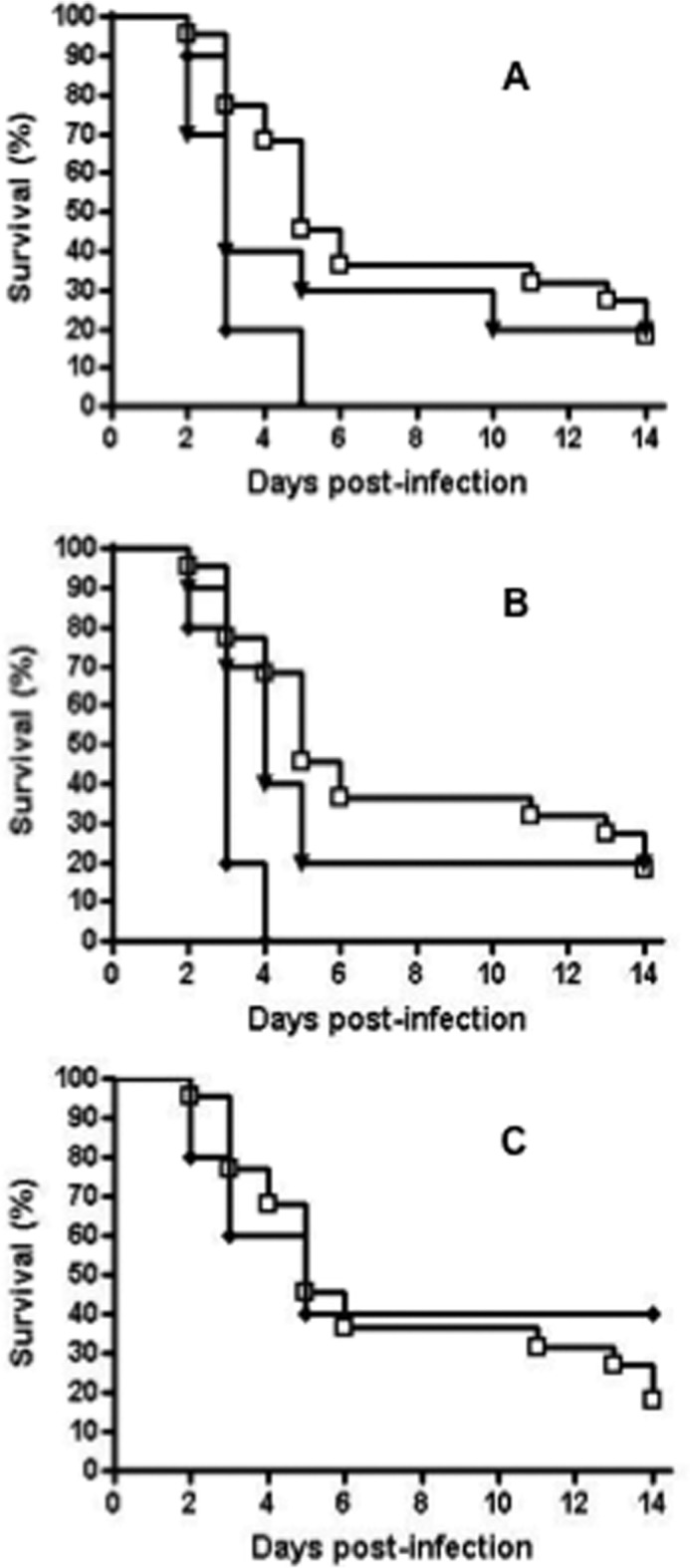
***C. glabrata anp1* and *mnn2* are hypervirulent.** *A,* survival curves for mice infected with *C. glabrata* 2001 (*open squares*), *anp1* (*closed diamond*), and *anp1*::*ANP1* (*closed triangles*). Intravenous infection with the *C. glabrata anp1* null mutant at 7 × 10^7^ cells/mouse resulted in a hypervirulent phenotype with 100% mortality within 5 days, compared with the wild-type strain at the same dose resulting in 20% survival over 14 days. This result is significant (*p* < 0.01, Kaplan-Meier log rank analysis). The reintegrant control restored virulence to wild-type levels with 20% survival over 14 days (*p* > 0.05, Kaplan-Meier log rank analysis). *B,* survival curves for mice infected with *C. glabrata* 2001 (*open squares*), *mnn2* (*closed diamond*), and *mnn2*::*MNN2* (*closed triangles*). Intravenous infection with the *C. glabrata mnn2* null mutant at 7 × 10^7^ cells/mouse resulted in a hypervirulent phenotype with 100% mortality within 4 days, compared with the wild-type strain at the same dose resulting in 20% survival over 14 days. This result is significant (*p* < 0.0001, Kaplan-Meier log rank analysis). The reintegrant control restored virulence to wild-type levels with 20% survival over 14 days (*p* > 0.05, Kaplan-Meier log rank analysis). *C,* survival curves for mice infected with *C. glabrata* 2001 (*open squares*) and *mnn11* (*closed diamond*). Intravenous infection with the *C. glabrata mnn11* null mutant at 7 × 10^7^ cells/mouse resulted in a wild-type phenotype with 40% survival over 14 days, compared with the wild-type strain at the same dose with 20% survival over 14 days. This result is not significant (*p* > 0.5, Kaplan-Meier log rank analysis. Groups (*n* = 22 wild-type cells; *n* = 10 for all mutants) of outbred CD1 mice were immunosuppressed with cyclophosphamide and infected with 7 × 10^7^ *C. glabrata* yeast cells via tail vein injection. Following infection, mice were weighed and observed daily and sacrificed at predetermined end points, *e.g.* 20% weight loss.

The *C. glabrata anp1* null was significantly increased in virulence with 100% mortality by day 5, compared with 20% survival over 14 days for the wild-type control (Fig. 7*A*, Kaplan-Meier log rank test; *p* < 0.01). Reconstitution of *ANP1* restored the phenotype to wild-type levels with 20% survival over 14 days (*p* > 0.05). Therefore, inactivation of *C. glabrata anp1* results in a hypervirulent phenotype in our mouse model. Similarly, the *C. glabrata mnn2* null was significantly increased in virulence with 100% mortality by day 4, compared with 20% survival over 14 days for the wild-type control (Fig. 7*B*, *p* < 0.0001). Reconstitution of *MNN2* restored virulence to wild-type levels with 20% survival over 14 days (*p* > 0.05). Therefore, inactivation of *C. glabrata mnn2* results in a hypervirulent phenotype in our mouse model.

In contrast, the *C. glabrata mnn11* null was unaffected in virulence with 40% survival by day 14, compared with 20% survival over 14 days for the wild-type control (Fig. 7*C*, *p* > 0.5). No *MNN11* reconstitution experiments were performed as virulence was wild type in the *mnn11* null. Hence, deletion of *MNN11* has no effect on virulence in our mouse model.

##### C. glabrata mnn2 Cells Are Hyperadherent to Endothelial Cells

**FIGURE 8.**
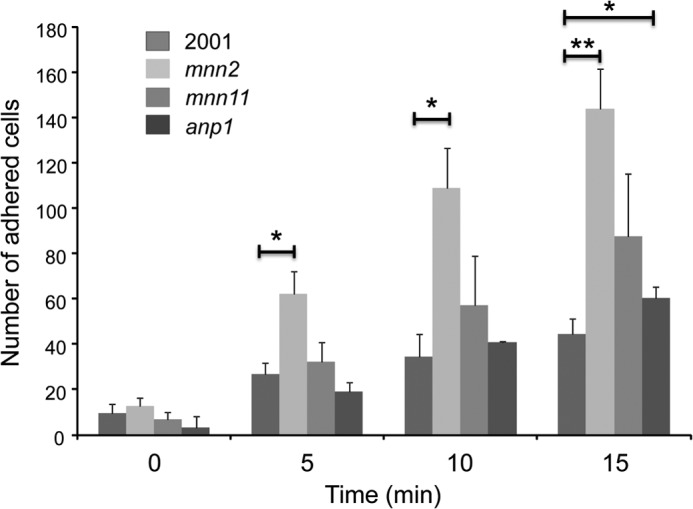
***C. glabrata anp1* and *mnn2* are hyperadherent to HEMC-1 endothelial cells.** Adhesion of *C. glabrata* 2001, *anp1*, *mnn2,* and *mnn11* to HEMC-1 endothelial monolayers under conditions of shear flow. *C. glabrata* 2001, *anp1*, *mnn2,* and *mnn11* cells were perfused through a flow chamber over an HMEC-1 monolayer, and adhered cells were counted at 0-, 5-, 10-, and 15-min time points. *C. glabrata* cells were suspended at 1.0 × 10^6^ yeast/ml in Hanks' buffered salt solution and perfused over glass slides coated with confluent HMEC-1 monolayers, mounted in a parallel plate flow chamber. Adhesion events were visualized from a random field of view (0.15 mm^2^) using a Zeiss Axiovert 200 M inverted fluorescence microscope. Images were then acquired over 15 min into a video file at 2 frames/min, and the total number of adherent cells/mm^2^ was recorded. All experiments were performed in a controlled environment chamber at 37 °C, and each experiment was repeated with three separate confluent endothelial cell slides on at least two occasions. The results shown are the pooled triplicate data from a representative experiment, and the standard deviation indicated. *, *p* < 0.05; **, *p* < 0.001, *t* test.

To determine whether the structural differences in the cell wall were able to affect the adherence capacity of the strains, and hence at least partially explain the differences in virulence observed, we employed a flow adhesion assay. *C. glabrata* cells were perfused through a flow chamber over a confluent layer of HMEC-1 endothelial cells for 15 min. Adhered cells were scored at 0, 5, 10, and 15 min for each strain, and the results from a representative experiment (three technical replicates) are shown in Fig. 8. *C. glabrata* adhesion to the endothelial cells was extremely rapid with adhesion noted for all strains after 5 min. *C. glabrata mnn2* was hyperadherent compared with *C. glabrata* 2001, with statistically increased levels of adhesion at all time points (5 and 10 min, *p* < 0.05; 15 min, *p* < 0.001; *t* test). *C. glabrata anp1* also showed statistically significantly increased adherence compared with *C. glabrata* 2001 at 15 min (*p* < 0.05, *t* test), although the increase was modest. *C. glabrata mnn11* was statistically unaffected in adherence compared with *C. glabrata* 2001 for all measured time points. Hence, both the *C. glabrata mnn2* and *anp1* endothelial cell interactions were altered as characterized by a hyperadherent phenotype *in vitro*, albeit modest in the case of *C. glabrata anp1* cells.

## DISCUSSION

Complementation analyses showed that the *C. glabrata ANP1, MNN2,* and *MNN11* encode functional homologues of the respective *S. cerevisiae* proteins. Therefore, we predict that both *C. glabrata* Anp1 and Mnn11 are α-(1,6)-mannosyltransferases, whereas Mnn2 functions as an α-(1,2)-mannosyltransferase (26). As anticipated, inactivation of each gene resulted in phenotypic consequences suggestive of changes in cell wall structure and integrity.

To investigate the structural basis for these phenotypic changes, we compared the mannans in each mutant to wild-type *C. glabrata*. All three mutants had mannans that exhibited lower molecular weight than mannan isolated from wild-type cells. The 48% reduction in molecular weight of *C. glabrata mnn2* demonstrates that this mannan contains the fewest mannosyl repeat units of all strains tested, which we suggest is due to an inability of the mutant to catalyze the addition of α-(1–2)-mannose residues to the α-(1–6)-linked mannosyl backbone resulting in long unbranched mannans. This is in agreement with a lack of α-(1–2)-mannose residues seen in an *S. cerevisiae mnn2* strain (68). In contrast, the *C. glabrata anp1* and *mnn11* strains display a less dramatic decrease in their molecular weight (degree of polymerization). Although their mannan backbone chains are shorter, α-(1–2)-mannose residues can still attach. Thus, the overall molecular weight of the *anp1* and *mnn11* mannan polymers is higher than that observed for *C. glabrata mnn2* mannan.

The Alcian blue assay showed that inactivation of *MNN2* resulted in a 75% reduction in mannosyl phosphorylation compared with wild-type cells, whereas the relative reduction was only 50% when *ANP1* or *MNN11* was deleted. The mannosyl phosphorylation that was observed is likely due to attachment of phosphomannan to either the *N*-linked core structure or *O*-mannan. However, the lower level of mannosyl phosphorylation in *C. glabrata mnn2* cells indicates that the α-(1,2)-mannose side chains harbor mannosyl phosphorylation linkage points, as seen in *S. cerevisiae N*-glycans (69) and similar to what has been reported in *C. glabrata N*-glycan structural studies (17, 18). The ^1^H NMR spectrum of mannan extracted from *C. glabrata mnn2* confirmed the lower level of mannosyl phosphorylation in this strain.

**FIGURE 9.**
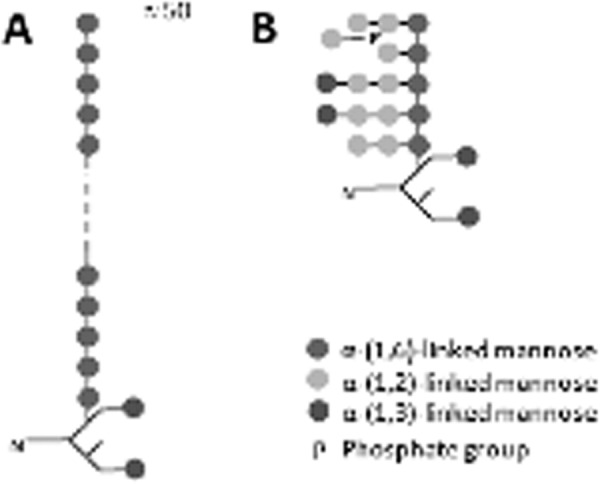
**Predicted mannan structures of *C. glabrata anp1*, *mnn2,* and *mnn11*.** *A,* predicted structure of the *C. glabrata mnn2* mannan as essentially a linear α-(1,6)-mannan with minimal α-(1,2)-linked side chain branching, and lacking the majority of mannosyl phosphorylation. *B,* predicted structure of *C. glabrata anp1* and *mnn11* mannans. These contain a shorter α-(1,6)-mannan backbone; however, they retain secondary and tertiary side chain branching and some mannosyl phosphorylation. The precise structure of the secondary and tertiary branches is not known and is predicted from the mannan structural work described herein.

Our results indicate that *C. glabrata* 2001 has a mannan structure composed of α-(1,6)-linked mannosyl backbone that has α-(1,2)-linked mannosyl side chains that are the principal site of mannosyl phosphorylation and that some of these side chains terminate in α-(1,3)-mannose residues. In *mnn2* cells, this is replaced by a structure that lacks α-(1–2)-linked mannosyl side chains and exhibits severely depleted levels of phosphomannan (Fig. 9*A*). Whereas *anp1* and *mnn11* cells retain α-(1–2)-linked mannosyl side chains and a greater degree of phosphomannan, they have shorter α-(1–6)-linked mannosyl backbones (Fig. 9*B*). These structures are in broad agreement with the structures recently described in three different *C. glabrata* strains (20). Additionally, these overall changes in mannan structure very likely reflect differential glycosylation of individual proteins, many of which would have phenotypic implications. Functional analysis of these changes could be extremely revealing.

Changes in *C. albicans* mannan structure dramatically impact upon cell wall integrity, virulence, and activation of innate immunity (12, 36). Here, we have shown that deletion of *C. glabrata* mannosyltransferases again impacts cell wall integrity. We have previously shown that inactivation of *C. glabrata ACE2*, although not directly involved in modulating mannan structure, had a profound impact on the cell wall and secretome that results in massive overstimulation of host innate immunity and increases virulence almost 200-fold (48, 70, 71). We therefore sought to determine the impact of Anp1, Mnn2, and Mnn11 on virulence. In a mouse model of systemic candidosis, both the *C. glabrata anp1* and *mnn2* mutants were hypervirulent compared with wild-type *C. glabrata* 2001, whereas *C. glabrata mnn11* exhibits wild-type like virulence. *C. glabrata ace2* cells form large clumps, and hence it is possible that death is attributable to obstruction of the vasculature. However, in the experiments described here, mice were infected with single cells (*C. glabrata anp1*) or very small cellular aggregates (*C. glabrata mnn2* and *mnn11*, *n* <5) clearly demonstrating that increased virulence can occur in the absence of vascular occlusion. We predict that a septic shock-like response occurs in the *C. glabrata anp1-* and *mnn2-*infected mice, as has been observed in mice infected with other hypervirulent *C. glabrata* mutant strains (48, 72). Certainly, the balance between pro- and anti-inflammatory responses is important in many fungal infections (73). Potent activation of pattern recognition receptors can lead to septicaemia and rising levels of pro-inflammatory cytokines such as TNF-α and IL-6, which can reflect the balance between inflammatory responses (74, 75). Cytokine analysis of the serum 24 h post-infection (four mice per group) was conducted to investigate this; however, the variation in absolute cytokine levels between mice was too high to allow comparison within such a small cohort. Hence, we have been unable to confirm or refute this prediction.

Another issue with respect to the virulence characteristics of *anp1* and *mnn11* strains needs to be addressed. In *S. cerevisiae*, Anp1 and Mnn11 are both members of the mannosyl polymerase II complex and either contribute to or exhibit α-(1,6)-mannosyltransferase activity (76, 77). Our data strongly suggest that this function is conserved in *C. glabrata*. So the question arises, why do strains that lack components of the same enzyme complex exhibit different virulence levels? Especially as we have shown that the outer chain mannan structure is similar in *C. glabrata anp1* and *mnn11* strains (Fig. 9). To address this, we sought to determine whether variations in the ability to elicit immune activation *in vitro* or adherence to endothelial cells, both important mediators of the host-fungal interaction, could explain this variation. *mnn2*, *mnn11,* and *anp1* cells were hyper-elicitors of TNF-α production from RAW264.7 cells compared with *C. glabrata* 2001 (supplemental Fig. 1), suggesting that they are all capable of overactivation of innate immunity, at least *in vitro*, and hence this cannot explain the virulence difference.

The ability to adhere to cells is a prerequisite for tissue penetration, invasion, and disease progression, and many of the proteins that mediate this interaction are, or are likely to be, glycosylated (78–81). The crucial role of adhesion in *C. glabrata* pathogenesis has been demonstrated by the observation that the glycosylphosphatidylinositol-linked aspartyl proteases encoded by *YPS1-11* are essential for virulence (78–81). We therefore sought to determine whether the differences in virulence could be explained by differences in adherence. Most reported studies of *Candida* adhesion employ static assays where the fungal cells remain in prolonged contact with cultured monolayers (82–84). Flow assays more closely mimic the passing and brief interactions that *Candida* cells have with endothelial cells, under conditions of shear stress and flow that occur in blood vessels (64, 85). In this study we compared the adhesion capacity of *C. glabrata* 2001, *anp1*, *mnn2,* and *mnn11* strains to endothelial cells under conditions of shear flow. In this flow adhesion assay, only *C. glabrata mnn2* cells were convincingly hyper-adherent to endothelial cells, compared with the parent *C. glabrata* 2001 strain, although *anp1* cells were also statistically increased in their ability to adhere to HMEC-1 cells. Hence, there is no distinct difference in this crucial aspect of the host-pathogen interaction that can explain the difference in virulence seen between *anp1, mnn2,* and *mnn11* cells. It remains to be elucidated what, if any, other structural differences there are between *anp1/mnn2* and *mnn11* cells and how this subsequently affects the interaction with the host. However, it is possible to speculate that the glycosylation of specific adhesins may influence these interactions significantly (78–81).

Finally, we have shown that inactivation of *C. glabrata ANP1* and *MNN2* results in an increased ability to cause disease, whereas loss of *MNN11* has no impact on virulence. This is in contrast to *C. albicans*, and other fungi, where changes in the external glycan structure often result in attenuation. Hence, it is not prudent to draw predictions of what we may expect to observe in *C. glabrata* based upon studies in *C. albicans. C. glabrata* and *C. albicans* are not particularly closely related phylogenetically (57), and it is not surprising that they have different virulence traits. It is vitally important, with the growing incidence of this pathogen and its innate resistance to many currently available antifungal drugs, that we dissect virulence attributes directly in *C. glabrata*.

## Supplementary Material

Supplemental Data

## Footnotes

3 The abbreviations used are:

GPC/MALLS: gel permeation chromatography-multiangle laser light scattering
r.m.s.: root mean square.
