# Supplementary material for: Differential Virulence of Candida glabrata Glycosylation Mutants
Source: J Biol Chem. 2013 May 28;288(30):22006–18. doi: 10.1074/jbc.M113.478743 (PMC3724654; doi:10.1074/jbc.M113.478743)
Supplement: Supplemental Data [file supp_288_30_22006__index.html]

Differential Virulence of Candida glabrata Glycosylation Mutants — Candida glabrata Glycosylation Mutants — Supplemental Data 

# Differential Virulence of *Candida glabrata* Glycosylation Mutants

## 

**Files in this Data Supplement:**

- Supplemental Figure S1
