# Supplementary material for: Differential Virulence of Candida glabrata Glycosylation Mutants
Source: J Biol Chem. 2013 May 28;288(30):22006–18. doi: 10.1074/jbc.M113.478743 (PMC3724654; doi:10.1074/jbc.M113.478743)
Supplement: Supplemental Data [file supp_M113.478743_jbc.M113.478743-1.docx]

Supplementary Figure 1

***C. glabrata anp1, mnn2* and *mnn11* cells elicit significantly increased TNF-α production from RAW264.7 macrophages.** *C. glabrata* 2001, *anp1*, *mnn2* and *mnn11* cells were exposed to RAW 264.7 macrophages at a ratio of 30:1 fungi:macrophage. After washing to remove unbound fungi, macrophages were incubated for six hours at 37°C and supernatants were collected for TNF-α quantification by ELISA. Results (mean + standard deviation) are pooled triplicate data from three separate experiments. *C. glabrata anp1*, *mnn2* and *mnn11* show statistically significant increased levels of TNF-α elicitation compared to *C. glabrata* 2001 (*p* < 0.05, one-way ANOVA). Reintegration of *MNN2,* or *ANP1* partially restored TNF-α elicitation to wild-type levels (*p* > 0.05, one-way ANOVA).

Murine RAW 264.7 macrophages were seeded in 24-well plates at 2.5 x 10^5^ macrophages per well and grown overnight in DMEM supplemented with 10% (*v/v*) FCS, penicillin (100 U/mL) (Invitrogen, UK), streptomycin (100 ug/mL) (Invitrogen, UK) at 37°C, 5% CO_2_. Overnight cultures of yeast strains grown in the appropriate media were washed three times with sterile PBS (137 mM NaCl, 2.7 mM KCl, 8 mM Na_2_HPO_4_, pH 7.3) counted, and resuspended at 1.5 x 10^7^ yeast/mL in RAW 264.7 media. Before infection, macrophages were washed three times with fresh media. Yeast was then added at a yeast:macrophage ratio of 30:1 and allowed to bind and be phagocytosed for 40 min at 37°C, 5% CO_2_. Macrophages were then washed extensively with PBS to remove unbound yeast and incubated in 0.5 mL of fresh medium for 6 h at 37°C, 5% CO_2_. All infections were done three times independently and triplicate wells used each time.

Goat anti-mouse polyclonal IgG TNF-α capture antibody (100 μg/mL, R&D Systems, UK) was diluted in sterile PBS to 0.5 μg/mL, and 100 μL aliquots added to each well of a high binding, 96 well flat-bottomed plate (Corning, USA) and incubated overnight at 4°C. The plate was washed three times in PBST (0.05% (*v/v)* Tween 20 in PBS) and 150 μL blocking solution (2% (*w/v*) BSA (fraction V powder), 0.02% (*w/v*) sodium azide in PBS) was added to each well and incubated for 1.5 h at 25°C and washed three times as above. PBS was used to dilute the recombinant mouse TNF-α protein standard (10 μg/mL, R&D Systems, UK) to 100 ng/mL and complete media used to dilute to 2.5 ng/mL; 50 μL was added to wells A1, A2, B1, B2 and a 1:2 serial dilution performed to wells A10 and B10. Samples were diluted appropriately with complete DMEM to a volume of 50 μL and aliquoted into test wells and incubated for 1.5 h at 25°C, and washed three times. Goat anti-mouse biotinylated polyclonal TNF-α (TNFSF1A) detection antibody (50 μg/mL, R&D Systems, UK) was added to each well at a concentration of 0.2 μg/mL (50 μL/well) and incubated for 1 h at 25°C and washed three times. Streptavidin-HRP conjugate (Biosource, California) was diluted 1:3000 in PBS and 50 μL added per well and incubated for 20 min at 25°C and washed three times. Tetramethyl-benzidine (TMB) liquid substrate was added to each well (50 μL) and colour allowed to develop, the reaction was stopped by addition of 50 μL H_2_SO_4_ (1 M) to each well. Sample absorbance was measured at OD 450/580 nm using a 96 well plate reader (Multiskan Ascent, Labsystems, Finland). Results from a single representative experiment are presented as the averages and standard deviation of nine measurements, and a one-way (*An*alysis *o*f *Va*riance) ANOVA statistical test applied to determine the significance of observed differences.
